# Wireless Intelligent Sensors Management Application Protocol-WISMAP

**DOI:** 10.3390/s101008827

**Published:** 2010-09-28

**Authors:** Juan Carlos Cuevas-Martinez, Manuel Angel Gadeo-Martos, Jose Angel Fernandez-Prieto, Joaquin Canada-Bago, Antonio Jesus Yuste-Delgado

**Affiliations:** Telecommunication Engineering Department, University of Jaen, Alfonso X El Sabio 28, 23700 Linares, Jaen, Spain; E-Mails: jccuevas@ujaen.es (J.C.C.-M.); gadeo@ujaen.es (M.A.G.-M.); jcbago@ujaen.es (J.C.-B.); ajyuste@ujaen.es (A.J.Y.-D.)

**Keywords:** wireless sensor networks, fuzzy rule-based system, application protocol

## Abstract

Although many recent studies have focused on the development of new applications for wireless sensor networks, less attention has been paid to knowledge-based sensor nodes. The objective of this work is the development in a real network of a new distributed system in which every sensor node can execute a set of applications, such as fuzzy ruled-base systems, measures, and actions. The sensor software is based on a multi-agent structure that is composed of three components: management, application control, and communication agents; a service interface, which provides applications the abstraction of sensor hardware and other components; and an application layer protocol. The results show the effectiveness of the communication protocol and that the proposed system is suitable for a wide range of applications. As real world applications, this work presents an example of a fuzzy rule-based system and a noise pollution monitoring application that obtains a fuzzy noise indicator.

## 1. Introduction

Many recent studies have focused on various aspects of wireless sensor networks (WSNs) [1]. These networks are composed of the following: A significant number of sensor nodes, which consist of a processing unit with limited computational capability and memory; sensors, a wireless communication device; and a limited power source. Although sensor nodes have strong constraints on their energy resources and computational capacity, they can be used for continuous sensing, event detection, event identification, location sensing, and local control of actuators [2]. As a result, the range of applications of WSNs is very wide, and includes environmental monitoring systems, intelligent agriculture, surveillance, health monitoring, traffic monitoring, and industrial control and monitoring.

In spite of the fact that sensor nodes have highly constrained resources (microcontroller, memory, battery, communications), numerous new functionalities have been proposed for WSNs. An example of an application adapted to WSNs is the integration of soft computing (SC) technologies, such as fuzzy logic, neuronal networks, and fuzzy rule-based systems (FRBSs) in sensor nodes [3]. FRBSs are considered to be knowledge-based systems in which the knowledge of the system is represented by a set of “IF-THEN” rules whose antecedents and consequents are composed of fuzzy logic statements (fuzzy rules). One of the main characteristics of these systems is the capacity to incorporate human knowledge by accounting for its lack of accuracy and uncertainty or imprecision.

On the other hand, WSNs represent an ideal scenario for distributed applications in which several application layer protocols have been proposed, such as sensor management protocols, task assignment and data advertisement protocols, and sensor query and data dissemination protocols.

Although considerable research has been devoted to different WSN applications, less attention has been paid to knowledge-based distributed applications for WSNs. In this sense, it is necessary to design an application layer protocol that allows the distribution of knowledge bases (KBs) among the sensor nodes, the collection of inferred data, the management of the sensor nodes, and the integration with other measurement applications.

Following our previous research on FRBS WSNs [4–6], this work proposes a new distributed system composed of multi-agent knowledge-based sensor nodes. The contributions of this work are as follows:

- We propose a distributed system in which every sensor node can execute a set of applications (FRBS, Collaborative FRBS, measurements, actions, *etc.*).
- We present a multi-agent structure, defined for the sensor nodes, that is composed of three agents: management, application control, and communication.
- We define and implement, in a real WSN, an application protocol that allows the proposed system, among other features, to distribute KBs to sensor nodes and collaborate among sensors to achieve a global network objective.
- We examine the performance of the application layer protocol in a real WSN.
- As real world applications, we present a FRBS application and a distributed WSN application for environmental noise pollution monitoring in an urban area.

The rest of the paper is structured as follows: the next section deals with related work and motivation. Section 3 describes the methodology that was used, including the multi-agent structure of the sensor nodes and a description of the application protocol. Section 4 shows the experimental results related to the application protocol performance, as well as two real-world applications of the proposed system. Finally, conclusions are presented in Section 5.

## 2. Related Work and Motivation

Communication capabilities are one of the main characteristics in sensor networks, and enable the networks to share information, knowledge, and sensing effort in sensor nodes. The introduction of intelligent capabilities into sensor networks requires the use of communication resources and their optimization. In this sense, Brignell [7] defined an intelligent sensor as one that modifies its internal behavior to optimize its ability to collect data from the physical world and to communicate the data in a responsive manner to a host system. Benoit *et al*. [8] presented a model of intelligent sensor systems that emphasized the ability to exchange knowledge with other actors. Karlsson [9] defined an intelligent sensor network as autonomous sensor nodes that exchange information, reason, and collaborate with each other. The specific application implemented should preserve energy resources and work as one unit when delivering fused and compiled sensor information to the end user. A new structural concept of intelligent sensors and networks with intelligent agents which provide communications elements was suggested by Mekid [10].

The past few years have witnessed a growing interest in the use of techniques based on SC to optimize the communication process between intelligent sensors. In this sense, the use of Artificial Neural Networks to discover redundant input data was proposed in [11]. Cui *et al*. [12] proposed a FLC algorithm to ensure that the sensor network attains a large coverage region and maintains dynamic *ad hoc* network connectivity between nodes. Shu [13] proposed a fuzzy optimization algorithm (FRBS) to efficiently adjust the sensor placement after an initial random deployment. A fuzzy logic control based QoS management scheme for WSANs was developed in [14]. It utilized a fuzzy logic controller inside each source sensor node to adapt the sampling period to the deadline miss ratio associated with data transmission from the sensor to the actuator. Averkin [15] showed a combination of embedded fuzzy logic and neural network models for information processing in complex environments. The most interesting aspect of this approach is the use of a WSN as a distributed computing environment for intelligent data processing methods. Srinivasan [16] presented a novel scheme for data-centric multipath routing in wireless sensor networks utilizing a fuzzy logic controller architecture at each node in the network to determine its capability to transfer named data packets based on its own battery power levels and the type of data being forwarded.

Marin-Perianu [17] proposed a distributed general-purpose reasoning (D-FLER) algorithm that uses fuzzy logic for fusing individual and neighborhood observations. Nakamura [18] described how information fusion is closely related to data communication in WSNs.

As described in [19], collaboration enriches functionality and enhances scalability and manageability of networked sensor systems, particularly in those comprised of a large number of heterogeneous sensor networks deployed over a large area. Effective collaboration may require that various sensor networks share synchronized data replicas. That paper proposed a novel data replication mechanism suitable for the limited bandwidth of sensor networks. The scheme uses neural networks for scheduling of replication. In both [20] and [21], a cooperating object was defined as a single entity or a collection of entities consisting of sensors, controllers (information processors), actuators, or cooperating objects that communicate with each other and are able to autonomously achieve a common goal.

In all of the previous works, the use of SC techniques was proposed to fuse or aggregate raw data in nodes to reduce its redundancy and transmit only the processed data. In [6] and [17], the use of a collaborative algorithm, based on embedding FRBSs into WSNs, was proposed to implement these fusion tasks. This algorithm achieves an improvement in the reliability, responsiveness, and accuracy of the sensors in WSNs.

Akyildiz [1] proposed a sensor network communication architecture that defines a protocol stack composed of five layers. Akyildiz described three possible protocols inside the application layer, including a sensor management protocol, a task assignment and data advertisement protocol, and a sensor query and data dissemination protocol.

To the best of our knowledge, although many application layer protocols for sensor networks have been defined and proposed, protocols for KBs and fussed data delivery that are adapted to sensor limitations in sensor networks remains a largely unexplored region. In this sense, the application protocol presented in this paper provides an appropriate solution to fill these requirements.

## 3. Application Protocol for Wireless Intelligent Sensor Networks

The main objective of our research is to design and implement a distributed system, based on a WSN, that allows the execution of a set of applications in the sensors (measures, actuations, FRBS, collaborative FRBS, *etc.*), updating of the sensor KBs, obtaining sensor data and inferences generated in the sensors, and managing the sensors.

The proposed system is composed of a computer connected to the Internet, where the host application runs, an access point or base station that allows the host application to transmit data to the sensors, a WSN, a FRBS adapted for execution in the sensors, and the communication protocol. The main functions of the components are as follows:

- Computer: edit the KBs (variables, fuzzy sets, and rules), access the sensor network, communicate with sensors, and monitor sensor state.
- Sensor network: allow sensors and computer to communicate. The network consists of an access point and a set of sensor nodes with sensing, data processing, and communicating capabilities.
- Application protocol: allow the elements of the system to communicate data and knowledge.

The proposed system was designed with the aim of minimizing sensor communication to prolong battery life. Therefore, the sensors operate in a work cycle in which they first execute the application (measure, actuate, infer its output, *etc.*), decide if it is necessary to connect with the computer or other sensors, and then are configured in a sleep mode. On the other hand, the protocol is independent of the platform, so it can be used in different sensors.

This work is focused on an application protocol that allows communication between the computer and sensors, as well as among sensors. The following sections show the multi-agent structure of the sensor software and a detailed description of the communication protocol.

### 3.1. Sensor Multi-Agent Structure

**Figure 1.**
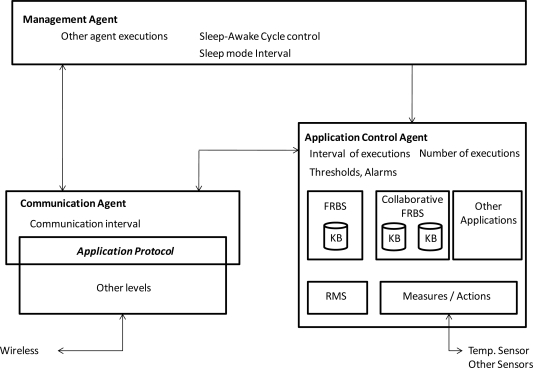
Multi-agent software structure in sensors.

The software of the sensors was designed based on a multi-agent structure, which is shown in Figure 1. The software is composed of three agents: management, application control, and communication. The management agent allows the system to turn up or turn down services or devices in the sensors and execute other agents. The control agent executes different applications in the sensor, such as measurements, actions, FRBSs, and collaborative FRBSs. Finally, the communication agent allows sensors to communicate with other sensors and with a computer connected to the WSN by means of an access point.

The following sections present the agent objectives and describe their functions.

#### 3.1.1. Management Agent

The management agent is executed when the sensor is powered up or when it returns from the sleep mode. Its main objective is the execution of other agents and the control of the sensor sleep-awake cycle, including the execution of the applications, program in sleep mode, duration of the sleep mode interval, and return to awake mode. Its functions are the following:

- Turn up or turn down sensor services and devices.
- Execute other agents.
- Control the sensor sleep-awake cycle and the sleep mode interval.
- Provide the sensor state (total awake time, number of executions, battery level, routing, *etc.*).
- Other maintenance functions.

#### 3.1.2. Application Control Agent

Executed by the management agent, the application control agent allows the sensor nodes to control the execution of different applications (take measurements of sensors such as temperature or humidity, actuate diverse actuators, infer an output in a FRBS, infer and collaborate in FRBS, *etc.*). The main functions of this agent are the following:

- Control the execution of different applications.
- Control the interval between consecutive application executions. This application interval may be different from the sleep mode interval.
- Control the schedule and the number of application executions.
- Update KBs in FRBS applications incrementally or completely.
- Schedule the way in which the sensor nodes notify their measurements or inferences (instantaneous values, average value in a period, alarms or values out of a range defined by thresholds, periodic values, *etc.*).
- Provide the agent state.
- Manage the persistent storage for other agents and applications.

#### 3.1.3. Communication Agent

The communication control agent incorporates the application protocol, which allows sensors to communicate with other sensors, neighboring sensors, and with a computer connected to the WSN by means of an access point. The main functions of this agent are the following:

- Communication with other sensors and the computer.
- Control the interval between consecutive communications with the computer. This communication interval may differ from previous intervals (sleep mode interval and application execution interval).
- Reception of commands from the computer. The set of commands and responses used in the application protocol is shown in Section 3.2.
- Transmission of measurements, inferences, and alarms (*i.e*. values of variables out of range defined by thresholds) that are generated in the sensor.
- Reception of KBs used by FRBSs and collaborative FRBSs.
- Transmission of alarms to a collaborative group, *i.e*. the set of neighboring sensors that are working in a collaborative FRBS application.
- Control the power transmission, taking into account factors such as the quality of the reception messages from the neighborhood and the battery level.
- Provide the agent state.

### 3.2. Protocol Description

The protocol was developed to fit in the application layer, and was implemented on Sun SPOTs [22] and on a computer. With this protocol, it is easy to transmit entire or partial KBs (variables, fuzzy sets, *etc.*). The KBs are generated and edited on the PC by a XML coded file, parsed by the host application, then transmitted to the sensors and executed there.

#### 3.2.1. General Features

This protocol has encouraged the versatility of its services to make sensors capable of achieving a wide range of tasks, which was one of the conclusions shown in [23]. According to [2], the main purpose of this protocol is to provide an Application Service Interface (ASI) to manage the problems generated with applications that access every low-level system of the device.

#### 3.2.2. Protocol Services

This protocol is agent oriented because its services are produced and consumed by the agents running inside the sensor, the neighbors, or the base station. These services were divided among three classes: sensor management, FRBS support and KB distribution, and alarm handling.

Sensor management services allow agents to send or receive configuration parameters or commands to control the sensor. Services that cover the second class can set up a new KB in a sensor or modify it with few transmissions. The third class allows agents to set up alarms and notifications that will be activated when some parameters exceed established thresholds. The services are explained in further detail in the following paragraphs.

Sensor management services:

- Get resource: With this service, any parameter of an agent or test probe can be requested.
- Set resource: This service is usually activated by a base station. It changes the value of any agent parameters, such as time to be idle and times to check values from a probe. The behavior of the agents is controlled through this service.

FRBS support and KB distribution services:

- Set KB: A new KB is sent to the sensor and stored for future use.
- Get KB: The sensor can send the KB to other neighbors or the base station to refresh their data.
- Get FRBS Result: Obtain the result of the FRBS.
- Set Rule: Each fuzzy rule can be accessed independently to adapt the FRBS to the environment.

Alarm services:

- Set Alarm: Establish a new alarm condition with the action to accomplish and the threshold for the monitored parameter.
- Alarm: An alarm beacon is broadcasted to inform the base station and the neighbors about the alarm condition in the sensor.

#### 3.2.3. Protocol State Machine

**Figure 2.**
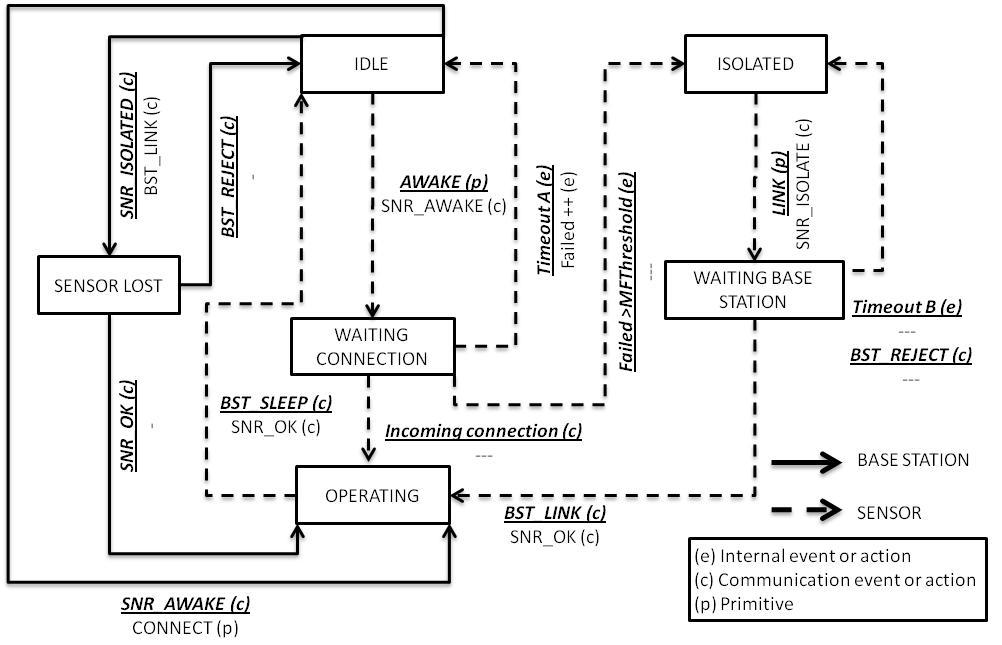
Protocol state machine.

The state machine is needed to keep track of several parameters to support the different services provided by the application protocol in order to avoid unnecessary transmissions as possible. Figure 2 presents the state machine, using boxes to represent states and arrow lines for transitions. For simplicity, not every possible minor transition is shown.

As shown in Figure 2, both the sensor and the base station share some states. Transitions in the base station are noted as solid lines, while dashed lines indicate sensor state changes. Each transition is labeled with additional information, including event type (bold text above the line) and action taken (text below the line). The kind of event and action is shown with a letter in brackets. Internal events can be produced by timers, battery charge, FBRS results, and values from probes. Communication events or actions always imply the sending or receiving of data by radio. Primitives are interface functions called by the sensor agents to access services. A summarized review of the states is presented below.

State description:

- Idle: For sensors, the idle state is also called sleep mode, and keeps only minimal system processes active and switches off most of the devices. In a base station, this is a listening mode.
- Isolated: The sensor does not have a base station to work with or has lost contact with it.
- Sensor lost: This state exists only in the base station, and is active when a SNR_ISOLATED is received. If the sensor belongs to the base station, it sends a BST_LINK message; otherwise, BST_REJECT is sent.
- Waiting base station: When the primitive Link is executed, a SNR_ISOLATED message is sent and the sensor goes into this state. If the sensor receives the BST_LINK message from a base station, it become active and starts working. If only BST_REJECT messages are received or the timeout B triggers, the sensor goes into the isolated state again.
- Waiting connection: The sensor has executed the primitive Awake and waits for incoming requests or commands from the base station.
- Operating: In this state, sensors and base stations exchange messages and run their internal applications to accomplish their tasks.

Primitives:

There are two types of primitives: confirmed and unconfirmed. Confirmed primitives have four steps, including request, indication, response, and confirm. Unconfirmed primitives do not need a response and confirm, so they only have request and indication. The type of primitive is distinguished by a ‘C’ or a ‘U’ in the descriptions below.

- Link (*maxhops*), C: Request a base station to form part of its WSN. Links send a broadcast message with a maximum number of hops, *maxhops*. This parameter is controlled by the communication agent.
- Command (*cid*, *agent*), C: Requires the execution of command *cid* by the desired *agent*.
- Request (*rid*, *agent*), C: Requires or sets some information of the desired *agent*.
- Awake (*timeoutA*, *maxhops*), U: The primitive broadcasts an “I am awake” beacon that informs that it is ready to receive a request. Like Link() primitive, Awake() has a *maxhops* parameter.
- Alarm (*aid*, *atime*), U: Sets an alarm of *aid* type produced at *atime*.

#### 3.2.4. APDU Structure

The Application Protocol Data Unit (APDU) is defined as a message in our development. There are three types of messages: commands, requests, and responses. All are identified by a 6 bit field in the APDU. Commands and requests have a very clear function defined by its identifier. Commands usually do not need payload data, while requests always need to be complemented by the payload content. Responses can carry payload data to add information about an action taken or an error, but this is optional.

As can be seen, the APDU carries information that breaks the layer isolation proposed by the ISO. However, this is necessary to achieve greater levels of accuracy in power estimation for transmission and application execution. With this information, the host application and other sensors can know if the sensor will be able to send further information or execute the installed applications in the future. We also know the current efforts in power optimization in the MAC layer, but this is under study and is not standardized yet. All of different types of messages will be listed briefly in Table 1.

#### 3.2.5. Protocol Procedures

Protocol procedures are the actions taken when a primitive is executed at any of its steps. These procedures make decisions based on the fact that they are being executed on an isolated sensor. Thus, all of the procedures tend to minimize communications and processing to optimize battery usage. The most important procedures used are detailed below:

- Priority management: All messages have a 2 bit field that stores the four priority levels, from 0 (lowest) to 3 (highest). When a message is received by a sensor, the priority is checked and the processed only if the battery level is enough for that priority level; otherwise, the message is discarded. For priority level 0, if the battery is below 75% the message is discarded; level 1 allows responses for a battery level down to 50%, level 2 allows responses down to 25%, and level 3 messages are always processed.
- Awake time estimation: In the current implementation, this is the estimate of the best time to get a result from the FRBS running in the sensor. This will be upgraded to a more complex system based in FRBS in the future.
- Base station detection: This is one important task that the protocol does itself. This procedure allows the sensor to be in movement or be aware of hardware failures in the base station. When a sensor does not have any base station set in its working parameters, it goes into the isolated state and must broadcast a SNR_LINK command to require a new one. If a sensor does not receive an incoming message after a certain number of times (*MFThreshold*), it assumes that it is isolated. This process uses the primitive Link. The parameters *maxhops* and *MFThreshold* have been extracted from the experiments shown in Section 4.
- Request control: All of the responses that should be transmitted must have the same sequence number as the request which originated them.
- Power and quality information: Every message sent by a sensor must contain information about the current charge of its battery and the power level used to transmit it. Moreover, the base station must send to a sensor the quality level measured for incoming messages broadcasted by sensors.

Acknowledgment messages are not present in the protocol, with the exception of the sensor_ok message that can be seen as an acknowledgment in some scenarios. Every request has a response, but the sending side does not know if the message reaches the other side.

### 3.3. WISMAP Advantages

The advantages associated with the use of the approach presented in this paper are closely related with the utility contributed by:

- The collaborative FRBS embedded into the sensor nodes, which can be summarized in:
  - An improvement in the reliability, responsiveness and accuracy of the sensor node behaviors.
  - A decrease in the amount of data to be transmitted to neighbor nodes.
  - To give support to the minimization of sensor failures and communication errors.
- The wireless intelligent sensors management application protocol presented, which can be summarized in:
  - A service interface, independent of the platform, which can be used with different sensor technologies.
  - To give support to a complete o incremental update of KBs.
  - Versatility in the way in which the sensor nodes notify their measurements, results or inferences.
  - To give support to minimize communication and processing in order to optimize battery usage.
  - To give support to new types of probes, actuators or sensors due to the open data format and hierarchical labeling system used in the protocol.
- The multi-agent architecture presented, which can be summarized in:
  - To give support to future applications. This architecture provides a new framework for sensors, which embeds sensor and application management, FRBSs, communication and storing.
  - Completely open architecture based on free development tools.
  - Task and alarm scheduling without any re-coding of installed applications.

## 4. Results

This work follows our previous research on the integration of FRBSs in WSNs. In [6] we showed the structure of two knowledge-based systems (FRBS and collaborative FRBS) that were designed to be executed into a sensor, the first version of the KBs distribution protocol, and different ways to integrate data and knowledge. In the results we showed the knowledge-based sensor performance (execution time and consumption of the sensor due to the FRBS systems) and the evaluation of the collaborative scheme showing the effects of collaborative FRBS sensors on a modeling system of pests in the culture of the olive tree. Moreover, a comparative analysis of the FRBS sensor behavior, using its control surfaces, was shown in different situations.

The application protocol and the multi-agent structure proposed in this work allow the integration of several applications inside the sensor. Therefore, this work presents a noise pollution monitoring application in order to show that the proposed system not only supports knowledge-based applications, but also measurement applications.

The following sections show the experimental platform that was used in the experiments, the results of data distribution and robustness tests including the power consumption due to communication, and a noise pollution monitoring application that was executed in the application control agent.

### 4.1. Experimental Platform

The sensor nodes used in the experiments were Sun SPOTs nodes (Sun Small Programmable Object Technologies) [22] that have the following main characteristics: 32-bit ARM920T processor running at 180 MHz, 512 KB RAM, 3.7 V 720 mAh rechargeable battery, 4 MB flash memory, six analog inputs readable by an Analog Digital Converter, five general I/O pins, and an IEEE 802.15.4 compliant radio TI CC2420 transceiver [24].

**Figure 4.**
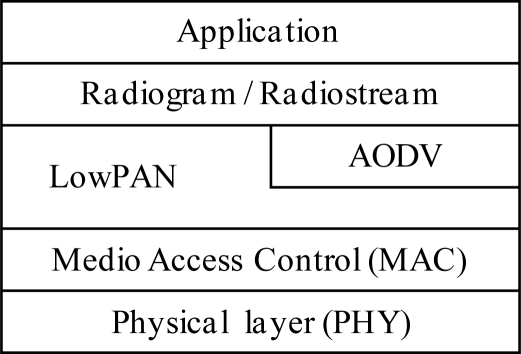
Sun SPOT network stack.

Figure 4 shows the specific Sun SPOT network stack protocol. A Sun SPOT’s radio chip is configured to accept all packets bearing this address or the broadcast address. Packets with other addresses are discarded. To provide routing, meshing, and fragmentation, the Sun SPOT stack relies on the LowPAN protocol [25]. Multi-hop connectivity can be accomplished using a sophisticated routing protocol for *ad hoc* networks, AODV [26]. Sun SPOTs feature a combination of LowPAN and AODV as network protocols. In the transport layer, the radiogram protocol and the radiostream protocol allow Sun SPOT applications to access the network.

### 4.2. Knowledge Base Distribution and Robustness Tests

The distribution of KBs to the sensors is a new feature of this protocol; therefore, it is important to determine how long it takes in real scenarios. Other important issues that a protocol has to achieve are robustness and power consumption, mainly in WSN. Different kinds of tests are described in the following sections to demonstrate the performance of the protocol in these tasks.

#### 4.2.1. KB Distribution in Real Scenarios

The real-world application where the KBs distribution was performed was described in a previous study [6]. We presented a knowledge-based WSN that concretely modeled a system of olive tree pests. The development of the olive tree fly is closely related to the temperature and humidity conditions of the environment. The suitability of insecticide treatment applications should be evaluated if the risk of the plague’s appearance in an area surpasses a threshold level.

The KBs were designed previously using an XML file that can have any number of variables, fuzzy sets, or rules; this file is validated with a Document Type Definition (DTD). Nevertheless, this file is not sent to sensors due to the presence of characters unrelated to later processes in the sensors. Therefore, this file is parsed in the host before sending it. The format is similar to a binary serialization of the values of interest to the KB, such as variable ids or point coordinates.

Five experiments were completed in the knowledge-based WSN, one for each of the five WSN layouts. The first WSN used only one Sun SPOT, just the final recipient, while the others used 2 more intermediate sensors for each experiment with a maximum of nine. In all cases the same base station was used, and the distance between motes was about 10 meters. The layout of the WSN was approximately linear, where every mote had only two motes within its range, the previous and the next in the line. The intermediate motes between the base station and the final mote work like mesh routers, and the final mote implements the application protocol.

**Figure 5.**
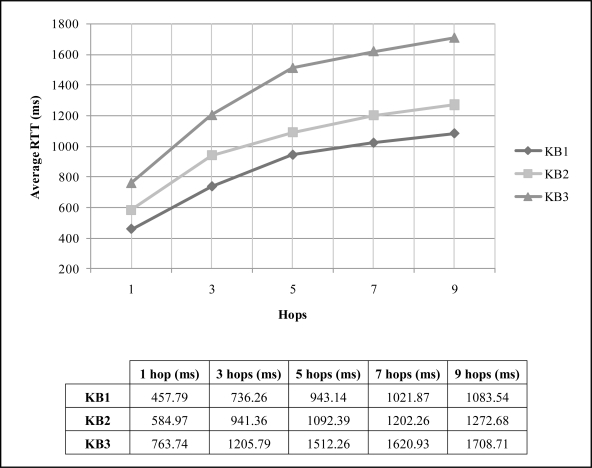
Average RTT values of the distribution of KBs.

In our experiment, we sent the three different KBs listed in Table 2. For each KB thirty tests were done. The results obtained were the round trip time (RTT) values in milliseconds which include the answer from the sensor for each experiment. Table 3 lists the lower and upper limits of the “confidence interval”. In 95% of the cases the “expected value” will be between the stochastic endpoints calculated for this confidence interval, but in 5% of the cases it will not be due to route re-calculation in the intermediate motes. Figure 5 shows the average values obtained for the transmission of every KB.

Figure 5 shows that the size of the KB is an important parameter and that the number of nodes makes the RTT grow as it was supposed. Nevertheless, the confidence interval is short because the deployed network is static and, once the route is calculated, it has no changes until the route expires in the intermediate nodes.

In order to prove the hypothesis that the delay increases with the number of hops and the size of KBs, a statistical analysis, a t-student test, was done to the average values obtained. The test checks if the differences in the delay average measures were significant for the different numbers or hops and sizes of the KB. A significance level α = 0.05 was applied for the test in question. Table 4 shows the results of two experiments; the first of them, “Experiment A”, tests the significance of the increase of the delay for the same number of hops with different KBs, whereas “Experiment B” tests the significance of the increases for the same KB with different numbers of hops. The sequences used for each experiment consist of 30 samples of values obtained for the number of hops and the KB listed in each row in the table. The “Test T” column shows the result of the test for the two sequences compared, where a plus sign (+) denotes a significant increase. As shown in Table 4, all tests are positive.

Taking into account these result, further efforts are needed in WSNs to ensure a reliable application protocol with minimum transmissions due to the significant increase of delay with number hops and size of the information. That goal can be achieved with an adaptive RTT estimation in lower layers (like transport layer) or by WISMAP, due to its modular structure where any parameter can be controlled by the final user.

#### 4.2.2. Robustness of the Protocol and Application Layer

The current version of the protocol, version 0.3, was tested in more than 130,000 cycles of awake and sleep, with no errors in the sensors or the base station, even with the host forced off in or with the sensors in motion. This number of cycles represents an average of 15 days of running time with sleep periods around ten seconds.

#### 4.2.3. Power Consumption

The test of battery consumption was implemented with an isolated sensor attached to a base station doing the awake process. In this scenario, the sensor broadcasts the SNR_AWAKE message; when the base station receives it, it sends a BST_SLEEP message because there are no pending queries for the sensor. When BST_SLEEP is received by the sensor, it sends the SNR_OK message and enters deep sleep mode. Therefore, with this test, the sensor has to send two messages and receive one, each 10 bytes long. The transmission power used is the maximum available for the sensor. The results are shown in Table 5 and shows that communications have to be minimized due to the power consumed. The average value is around 0.006357%, so a sensor with full battery can perform about 15,700 cycles, which represents almost 10 days with cycles of 60 seconds.

### 4.3. Noise Pollution Monitoring

One of the most important features of the protocol and the multi-agent system deployed on the sensor nodes is the versatility of all of the components. Therefore, it is possible to implement small applications inside a measurement manager of the Application Control Agent without changing the protocol. As an example, we implemented an application for the monitoring of environmental noise pollution in an urban area.

There is a growing interest in monitoring environmental pollution parameters in urban areas. Recent studies [27] have demonstrated that exposure to environmental noise increases the risk of hypertension, hearing loss, and sleep disorders, and that it negatively influences productivity and social behavior. Thus, European Directive 2002/49/EC requires member states to provide accurate mapping of noise levels throughout all urban centers with more than 250,000 citizens.

Some authors have proposed the deployment of WSNs to monitor noise pollution and create maps of noise levels [28–30]. The noise indicator used as a criteria for the assessment of occupational noise, according to ISO standard 1999 [31], and used to create noise maps is called equivalent continuous sound pressure level, *L_eq_*, and is defined as:

(1) $$L_{\mathrm{eq},T}=10{\text{log}}_{10}(\frac{1}{T}\int_{0}^{T}\frac{p\;{\left(t\right)}^{2}}{p_{o}^{2}}dt$$

where *p(t)* represents the root mean square instantaneous sound pressure produced by an acoustic wave and *p_o_* is a reference value corresponding to the minimal audible acoustic signal for a human at 1 KHz, 2 × 10^−5^ Pa. However, to simulate the frequency response of the human, *p(t)* is passed through a filtering stage, A-weighting, which is a commonly used frequency weighting that reflects the loudness perceived by human. Thus, we used the indicator L_Aeq,_ measured in decibels (dBA), which captures the A-weighted sound pressure level of a constant noise source over the time interval T, which has the same acoustic energy as the actual varying sound pressure over the same interval [32]. The A-weighting equivalent continuous sound pressure level is defined as:

(2) $$L_{\mathrm{Aeq},T}=10{\text{log}}_{10}(\frac{1}{T}\int_{0}^{T}\frac{p_{A}{\left(t\right)}^{2}}{p_{o}^{2}}\mathrm{dt}$$

**Figure 6.**
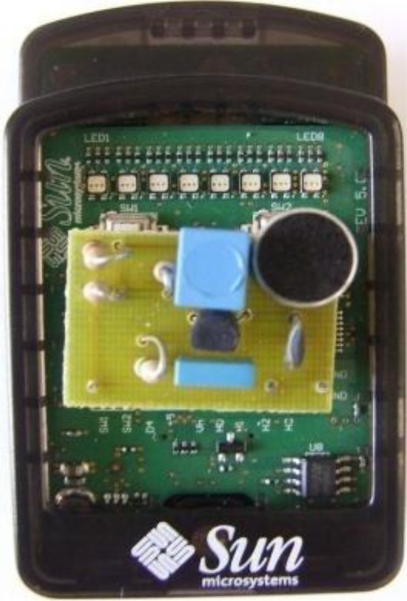
Sun SPOT node with the analog circuit.

To perform the experiment related to the measurement manager, we designed and implemented a simple analog circuit, equipped with an electret microphone, which was incorporated with a Sun SPOT sensor, as shown in Figure 6. In addition, we used a base station and a personal computer.

**Figure 7.**
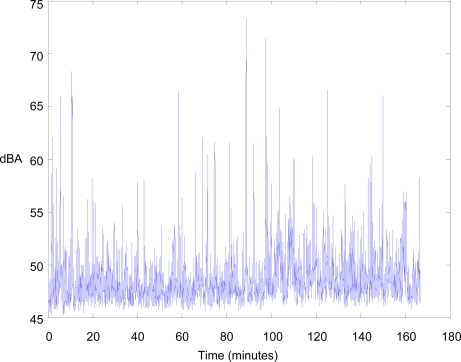
A-weighting equivalent noise level values measured by the sensor node.

The Sun SPOT sensor with the analog circuit was deployed close (about 10 meters) to an urban road. The application collects raw acoustic samples at a rate of 8 KHz, calculates the L_Aeq,T_ with a temporal granularity T of 125 ms (using 1,000 samples) each second, and transmits the value to the base station. Figure 7 shows the A-weighting equivalent noise level values measured by the sensor node for 166 minutes (from 4 p.m. to 6:45 p.m.). The values are generally around 48–52 dBA, which indicates that the noise pollution is moderate. Nevertheless, there are some times where the noise levels are higher; these correspond to passing vehicles.

**Figure 8.**
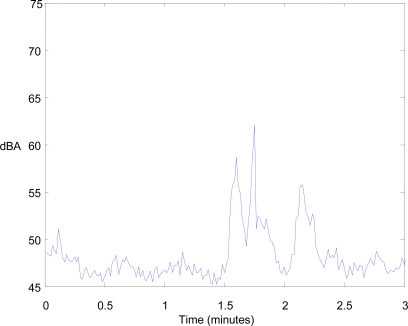
A-weighting equivalent noise level values measured by the sensor node during the first three minutes.

Figure 8 shows the A-weighting equivalent noise level values collected during the first three minutes of the experiment. There are three peaks, which represent three vehicles in transit, of which the first two were closer than the third. For the third, the period between when the sensor node detects the noise until it stops was around 18 s.

**Figure 9.**
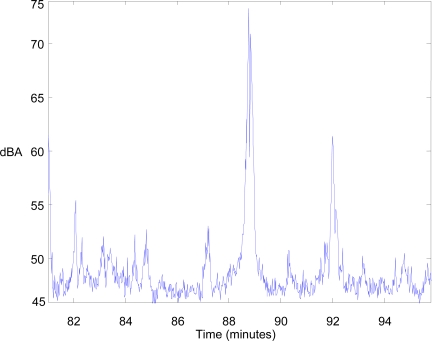
The highest peak of the A-weighting equivalent noise level.

Figure 9 shows the highest peak of the noise level that was measured. In this case, the noise was caused by a light aircraft which flew overhead.

The noise indicators defined by the European Commission directives can be calculated by the sensor nodes. However, the noise perception is affected by subjective factors and there is not a direct correlation between the indicators and the subjective perception of noise. The calculated noise levels are by no means adequate indicators for the effects of noise on humans. There are other factors that determine the way in which people perceive noise as, for example, the duration of the noise.

**Figure 10.**
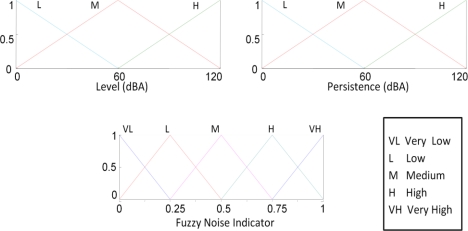
Membership functions of the inputs and output variables fuzzy sets.

In this way, we present a Fuzzy Noise Indicator (FNI) that allows sensor nodes to infer the degree of subjective noise annoyance. The proposed FRBS has two inputs (the A-weighting equivalent noise level value and its persistence in time calculated as the average of the last 10 values) and one output fuzzy variable (FNI). Figure 10 shows the KB variables and their membership functions, and Table 6 presents the set of rules used.

**Figure 11.**
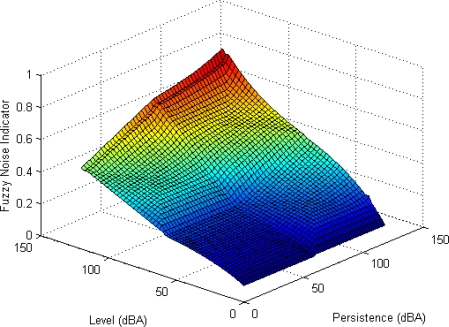
Input-output surface.

**Figure 12.**
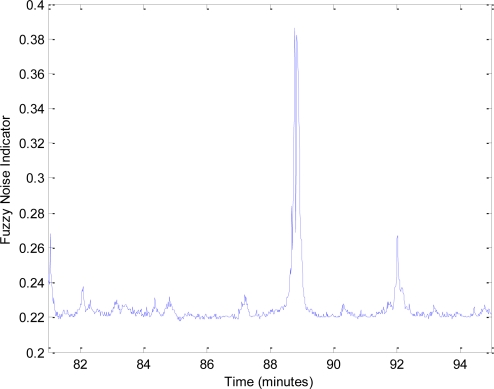
FNI for the noise data collection shown in Figure 9.

Figure 11 shows the input-output surface which relates the values of the noise level and its persistence with the FNI, and Figure 12 presents the inferred values for the noise data collection that is shown in Figure 9.

The use of this FNI can help to distinguish between situations with noise annoyance, characterized by a high level and persistence, and other situations less annoying. It is to stand out that using the FRBS, only the FNI values that surpass a threshold level are transmitted, reducing communication and battery consumption.

## 5. Conclusions

This work presented a distributed system adapted for WSNs where sensor nodes present a multi-agent structure that allows sensors to execute a set of applications (such as FRBS, measurements, and actions), manage sensor services, and communicate with other sensors and a computer. Communications are carried out using an application layer protocol that allows the nodes to transmit and receive information such as KBs, inferred or collected data, and alarms. Two real world applications of the system, a FRBS and a noise pollution monitoring system, were shown.

The noise pollution monitoring application presented in this paper, which was composed of the application, the multi-agent software, and the application layer protocol, represents a new monitoring opportunity that can be used to obtain maps of noise levels in time and space.

Moreover, the results show that it is possible to use this system in a wide range of applications, as well as the effectiveness of the application layer protocol. The distribution times of the KBs obtained in the real WSN were very short, and both throughput and delay showed similar values to the values obtained in previous network simulator experiments.

The multi-agent structure presented in this paper allows users to integrate different applications in a sensor node in an effective way. The definition of a service interface in the application layer allows new applications to be designed independently of hardware, memory, communications, sensors, and actuators, and access all of the components in a standardized way.

## Figures and Tables

**Figure 3.**
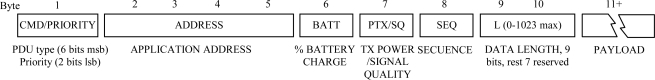
Protocol PDU.

**Table 1.** Protocol messages.

**Mnemonic**	**HEX code**	**Binary code**	**Description**
SNR_AWAKE	40 h	0100 00XXb	The sensor has woken up from sleep mode and is ready to accept queries from the base station
SNR_ISOLATED	44 h	0100 01XXb	The sensor has no a base station associated to it and requests a new one
SNR_OK	48 h	0100 10XXb	The sensor accepts the last message sent to it and informs that it has been executed properly
SNR_VALUE	4C h	0100 11XXb	The sensor returns the values requested by a GET_VALUE message
BST_LINK	04 h	0000 01XXb	The base station informs the sensor that it has been linked to that base station
BST_SLEEP	08 h	0000 10XXb	The base station commands the sensor to enter in deep sleep mode
BST_KB	10 h	0001 00XXb	This command loads a new KB into a sensor
BST_REJECT	0C h	0000 11XXb	The base station informs the sensor that it is not allowed to link to that base station
GET_VALUE	C4 h	1100 01XXb	The sensor is requested to send back some values from it (probes, agent parameters, *etc.*)
SET_VALUE	C8 h	1100 10XXb	This command establishes a new value of agent parameters, probes or actuators

**Table 2.** Knowledge base details.

	**Kb1**	**Kb2**	**Kb3**
Input variables	3	2	4
Output variables	1	1	1
Fuzzy sets	12	7	13
Rules	2	1	5
KB size (bytes)	332	492	740

**Table 3.** RTT distribution of KBs interval in which the probability of meeting the parameter “expected value” is 0.95.

**Hops**	**KB1 (ms)**	**KB2 (ms)**	**KB3 (ms)**
1	453.01–462.57	576.87–593.06	757.27–770.22
3	723.33–749.18	918.61–964.12	1184.8–1226.8
5	921.86–964.41	1067.23–1117.63	1482.5–1542.1
7	993.20–1050.50	1176.43–1228.19	1586.3–1655.6
9	1030.20–1136.91	1247.67–1297.75	1670.6–1746.8

**Table 4.** T-student test.

**Experiment A**					**Experiment B**				
First sequence		Second sequence		Test T	First sequence		Second sequence		Test T
N. of Hops	KB	N. of Hops	KB		N. of Hops	KB	N. of Hops	KB	
1	1	1	2	+	1	1	3	1	+
1	2	1	3	+	1	2	3	2	+
3	1	3	2	+	1	3	3	3	+
3	2	3	3	+	3	1	5	1	+
5	1	5	2	+	3	2	5	2	+
5	2	5	3	+	3	3	5	3	+
7	1	7	2	+	5	1	7	1	+
7	2	7	3	+	5	2	7	2	+
9	1	9	2	+	5	3	7	3	+
9	2	9	3	+	7	1	9	1	+
					7	2	9	2	+
					7	3	9	3	+

**Table 5.** Power consumption per awake cycle.

**Times**	**Battery charge**	**Power Consumption**	**Ratio per cycle**
0	97%	-	-
1020	90%	7%	0.006863
3952	70%	20%	0.006821
4314	68%	2%	0.005525
5572	60%	8%	0.006359
7020	51%	9%	0.006215

**Table 6.** Set of rules used.

***Fuzzy Noise Indicator***	***Persistence***			
***Level***		**L**	**M**	**H**
	**L**	**VL**	**VL**	**VL**
	**M**	**L**	**L**	**M**
	**H**	**M**	**H**	**VH**

VL: Very Low; L: Low; M: Medium; H: High; VH: Very High
